# The Bryophyte Flora of Vienna

**DOI:** 10.3390/plants12163002

**Published:** 2023-08-20

**Authors:** Harald G. Zechmeister, Michaela Kropik

**Affiliations:** Department of Botany and Biodiversity Research, University of Vienna, Rennweg 14, 1030 Vienna, Austria; michaela.kropik@univie.ac.at

**Keywords:** urban biodiversity, urban climate, urban planning, green infrastructure, mosses, liverworts, threatened species, conservation

## Abstract

The bryophyte flora of Vienna is documented only in parts. Old finds often appeared in publications about Lower Austria; only one study addressed the bryophytes of the inner city. Here, we present a bryophyte flora of Vienna, including historical reports and the results of recent investigations. From 1998 to 2023, we recorded 329 bryophyte taxa in Viennese urban territory. Fifty-six of these were liverworts, and 273 were mosses. Sixty-seven taxa are new for Vienna. Forty-nine taxa, given in historical studies, could no longer be found. If we also count these, 378 taxa occurred in Vienna to date. Of the current occurring bryophytes, 67 species have an endangerment classification. Rich in bryophytes were the dry grasslands of the Lobau, the oxbow lakes of the Lobau and the Prater, and large parts of the Wienerwald. But flat roofs and inner-city areas also showed more than 100 species. Compared to other European cities, Vienna is decidedly species-rich and highly responsible for some species in Austria. Reasons for this are the extensive green spaces and the pronounced climatic gradient from the sub-oceanic west to the sub-continental east of Vienna. Awareness raising for bryophytes we recommend in addition to the existing biotope protection.

## 1. Introduction

**Figure 1:**
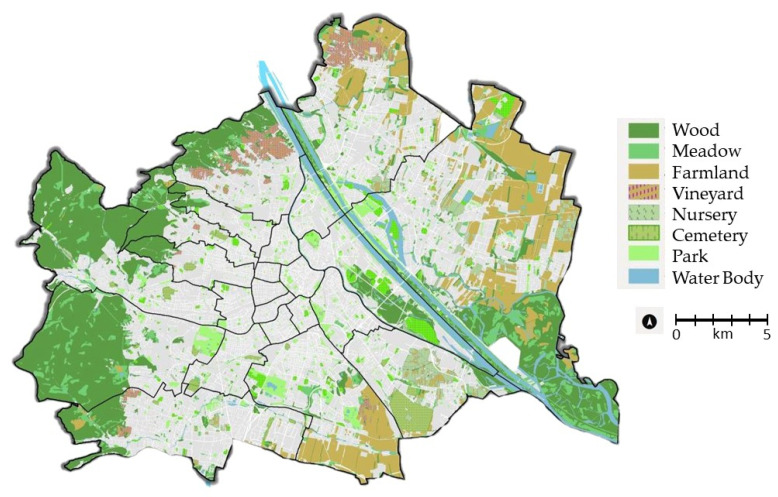
Vienna and its green spaces [21].

“Vienna is different”—this slogan of the Vienna city government also applies to the size of the green space in Vienna (Figure 1), which accounts for 31% (=128 km^2^) of the total city area [1]. In addition to numerous large parks, Vienna shares a National Park [2] and a UNESCO biosphere reserve [3], which is unique for a metropolis the size of Vienna. The high proportion of semi-natural vegetation suggests high species numbers in bryophytes.

**Figure 2:**
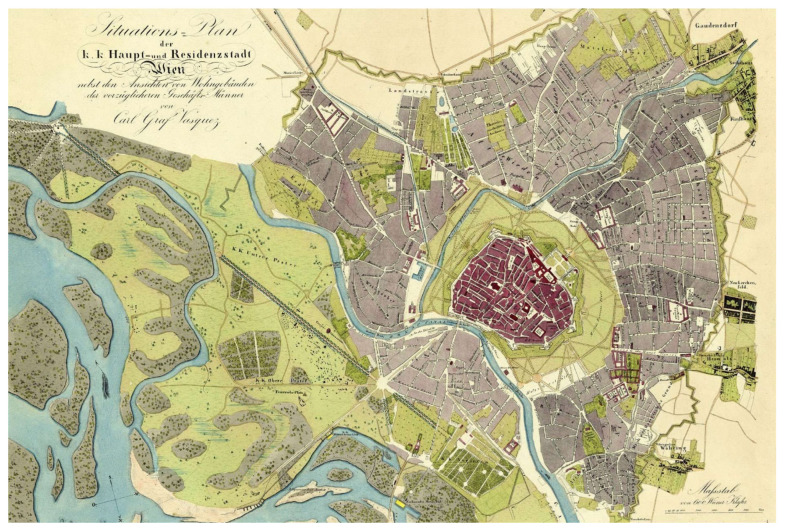
Vienna around 1830 (north below) [22].

Historical surveys of bryophytes indicate the high bryophyte diversity of Vienna [4,5]. Many of today’s city districts were villages on the outskirts of a capital city until the 19th century. Agriculturally used land lay between the surrounding settlements and the city. Until 1870, the Danube was an untamed river that passed north of the town in many arms, leaving numerous islands and floodplains between the river arms (Figure 2). Large areas (e.g., the so-called Glacis) lay between the city walls and the suburbs to provide a direct line of fire on any attackers. Many of these areas were investigated by the bryologists of the 18th and 19th centuries. They reported bryophyte finds in Vienna integrated into studies on surrounding Lower Austria: e.g., Jacquin [6], Welwitsch [7], Garovaglio [8], Pokorny [9], Poetsch [10,11], Reichardt [12], Neilreich [13], Juratzka [14], Höfer [15], Höhnel [16], Heeg [17], Matouschek [18], Onno [19], and Baumgartner (W, unpubl.). Although most of these formerly bryophyte-rich habitats have given way to densely built-up areas, near-natural habitats have been preserved, especially on the outskirts of the town. The inner city also shows a high structural diversity and, thus, diverse bryophyte species [20].

Today, bryophytes are arousing interest again in many people, be it from an architectural or artistic point of view. In many places, there are attempts to green urban infrastructure with the help of bryophytes, but mostly with little success in Central Europe [23]. Architects demand quick greening success, which the bryophytes cannot deliver. There is also little knowledge about which bryophyte species occur naturally in the urban environment of Vienna. The background of the present work is to expand knowledge about this and to improve the data situation on the biodiversity of Vienna.

## 2. Results

Overall, 378 bryophyte taxa (376 species and 2 additional varieties: 2 hornworts, 68 liverworts, and 308 mosses) occurred on Viennese urban territory if all historical reports are included (see Appendix A, Table A1).

During this study, we found 329 taxa (327 species and 2 varieties), including 56 liverworts and 273 mosses. Hornworts we could not find anymore. For 67 species (12 liverworts, 55 mosses), there are no records in the historical literature. They are the first records for Vienna, found during the current surveys and published here for the first time. Forty-nine species known from historic reports could not be reconfirmed and must be considered lost. Sixty-eight species are listed in the Red List of Lower Austria [24]. Nine are classified as threatened with extinction, and 17 as endangered.

### 2.1. Critical Taxa of the Historical Literature

Some historical reports are critical due to changes in taxonomy and nomenclature. However, it was not possible in most cases to consult the corresponding specimens and verify the data. The report of *Jungermannia michauxii* (today *Anastrophyllum michauxii*) in 1825 might have been the related species *Sphenolobus minutus* (Schreb. ex D. Crantz) Berggr., a species described later. *Pohlia filum* is also doubtful and was probably *Pohlia elongata*. However, an occurrence of *Pohlia filum* on the banks of the Danube at that time seems possible. *Schistidium confertum* is probably *S. brunnescens*, which we also found during this study. The historical report of *Warnstorfia fluitans* is most likely a misdetermination since this mire species could hardly occur in Vienna, which was free of *Sphagnum* species (also in former days), and we thus excluded it from the species list.

### 2.2. Critical Taxa of the Current Surveys

The introduced *Nogopterium gracile* was found only once in the garden of the Schloss Belvedere. This Mediterranean species might have reached its locality via substrate or plants. It is not assumed established to date, but we included it in the species list under this restriction. *Philonotis marchica* might have also come to Vienna via plant material. However, we found it several times, mainly in flower boxes and parks in downtown Vienna. An establishment there can be assumed.

### 2.3. Sites Particularly Rich in Bryophyte Species

The areas with the highest species diversity were in the Viennese part of the Donau-Auen National Park, at the Donauinsel, and in the Wienerwald. However, inner-city areas also had considerable species numbers.

**Figure 3:**
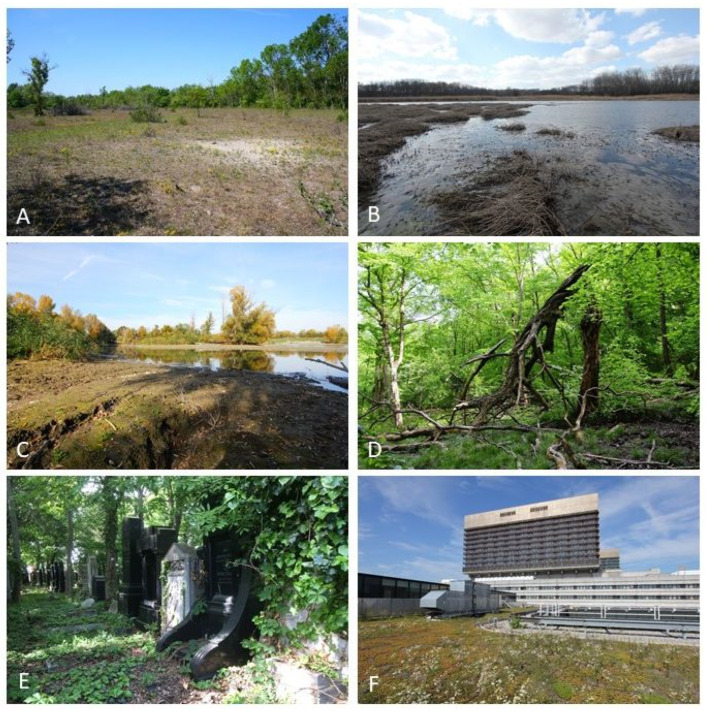
Habitats rich in bryophyte species in Vienna: (**A**)—Dry grassland in the Lobau (Kontrollerwiese, Donau-Auen National Park), (**B**)—Oxbow in the Lobau (Mittelwasser, Donau-Auen National Park), (**C**)—Floodplain at the Donauinsel, (**D**)—Virgin Forest Johannserkogel (Lainzer Tiergarten, Biosphere Reserve Wienerwald), (**E**)—Zentralfriedhof (Vienna Central Cemetery, Jewish part), (**F**)—Flat roof at the Vienna General Hospital (inner districts).

The so-called Heißländen of the Lobau (Figure 3A) deserve special mention: these gravel areas filled up by exceptional floods of the Danube correspond to edaphic dry grasslands. They are all located in the upper Lobau and harbor numerous bryophyte species that have become rare in Austria. In six of those Heißländen investigated, 98 species were found, including the rarities *Cephaloziella stellulifera*, *Entosthodon fascicularis*, *Mesoptychia turbinata*, *Microbryum floerkeanum*, *Microbryum starckeanum*, and *Aloina ambigua*.

Oxbow lakes in the Lobau and Prater (Figure 3B) are also species-rich sites: although the remnants of the formerly free-flowing Danube no longer have dynamic sections, these almost stagnant waters and their muddy banks, which are often overgrown with reeds, still harbor rare species, including *Amblystegium humile*, *Archidium alternifolium*, *Campylium polygamum*, *Fontinalis hypnoides*, *Riccia warnstorfii*, or *Ricciocarpos natans*.

Also, the loamy soils in the floodplain of the Donauinsel (Figure 3C) are a habitat for bryophytes. During extreme water levels of the Danube, opening the locks to the New Danube is a flood protection measure. A result of these floodings are deposits of loamy sands on the Donauinsel. These are colonized by pioneers of moist soils (e.g., *Physcomitrium patens*) soon after the water recedes. Subsequently, these soils dry out until the next flood, which often occurs years later. There, a dry grassland flora develops with sometimes rare elements, such as *Pterygoneurum lamellatum*, *Aloina brevirostris*, and *A. ambigua*. Over a length of 21.1 km, the Donauinsel offers a large number of these sites, which are rare throughout Austria.

The area of the Lainzer Tiergarten also offers important habitats, such as deciduous woodlands, litter meadows, wet meadows, meager dry meadows, spring horizons, and streams. Notable is the virgin forest remnant Johannserkogel (Figure 3D), hosting considerable deadwood volumes. Also, the Kaltbründlwiese crisscrossed by (coldwater) streams is rich in bryophytes. Overall, we found more than 100 species in the Lainzer Tiergarten—a high number for an area dominated by beech forests.

The variety concerning pH value in the Wienerwald allows the settlement of diverse soil and rock colonizers among bryophytes. Soil reactions in the Flyschwienerwald vary and range from slightly alkaline (e.g., Hermannskogel or Schutzengelberg area) to comparatively strongly acidic (e.g., in the Steinerne Lahn area) [25]. The streams in the Flyschwienerwald enable the occurrence of species, such as *Seligeria pusilla* and *Blindiadelphus recurvatus*, which are rare in the other parts of the Wienerwald. The part of the Wienerwald dominated by carbonate is small and lies southwest of Vienna. One of the rarest finds there was the occurrence of *Asterella saccata*.

Also, cemeteries and parks are habitats for bryophytes in the city. Of Vienna’s 55 cemeteries, the Zentralfriedhof (Figure 3E) is particularly significant concerning bryophyte diversity. Its Jewish part is especially species-rich (70 species) since it is highly diverse concerning structures. Except for the Botanical Garden, generalist species dominate the city’s parks, most of which are highly stressed by air pollutants due to traffic. But the parks in the west of Vienna often merge smoothly into the Wienerwald and are correspondingly richer in species.

Even the inner city and rooftops are bryophyte habitats (Figure 3F). The inner city of Vienna was studied by Hohenwallner [20,26], and the occurrence of bryophytes on the flat roofs of buildings from the turn of the last century was studied by Zechmeister [27]. More recent investigations on the Vienna General Hospital (Allgemeines Krankenhaus der Stadt Wien) brought further finds [28], such as that of *Rhynchostegium megapolitanum*. In general, the vegetation of the roof surfaces corresponded in part to that of primary dry grasslands. In total, around 100 bryophyte species were found in the densely built-up area of Vienna. The high species number shows that numerous bryophyte species conquer the inhospitable city without human intervention. They only need the necessary time and adequate structures.

### 2.4. Biogeographic Elements of Vienna Compared to Austria

**Figure 4:**
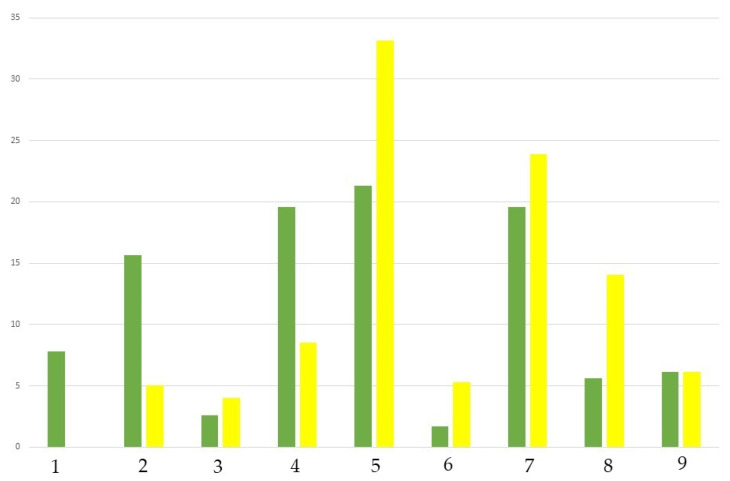
Categorization of taxa to biogeographic elements—comparison of the Austrian bryophyte flora (in green) with the bryophyte flora of Vienna (in yellow). 1—Arctic-montane, 2—Boreo-arctic montane (in tundra and coniferous forest zones); 3—Wide-boreal (from temperate zone to tundra), 4—Boreal-montane (main distribution in coniferous forest zone), 5—Boreo-temperate (in conifer and broadleaf zones), 6—Wide-temperate (from Mediterranean region to coniferous forest zone), 7—Temperate (in broadleaf forest zone), 8—Southern-temperate (in Mediterranean region and broadleaf forest zones), 9—Mediterranean-Atlantic (in Mediterranean region, and extending north in Atlantic zone of temperate Europe); Assessment of species to major biomes following Hill et al. [29]; Y-axis in %.

**Figure 5:**
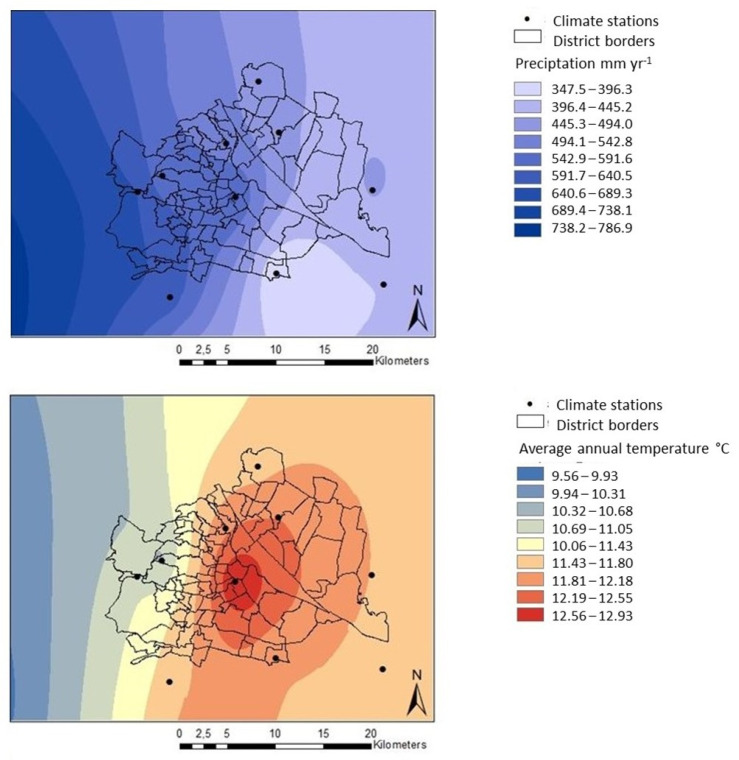
Precipitation and temperature gradients in Vienna; based on climate data of climate stations (black dots) in Vienna and Lower Austria from 2014–2017.

The comparison of the bryophyte flora of Vienna concerning biogeographic elements with that of Austria illustrates its diverging nature. The differences concerning the distribution of biogeographic elements are statistically significant (Chi-square test, *p* < 0.01): Austria has a high proportion of cool-temperate species (1–3 in Figure 4). In Vienna, boreo-temperate species dominate, and no arctic-montane species occur. Vienna’s high proportion of southern-temperate species, which have their center of distribution in the Mediterranean region, is also striking (Figure 4). This distribution of biogeographic elements reflects Vienna’s biogeographical position in the transitional area between the Pannonian and Alpine biogeographical regions. Concerning thermal radiation, Vienna has a (sub)Mediterranean influence. The urban heat island (Figure 5) causes the clustered occurrence of species adapted to a warm climate.

## 3. Discussion

The species diversity of bryophytes in Vienna is notable for a metropolis in the Pannonian climate, which is a harsh environment for bryophytes. It has several causes, first and foremost, the high proportion of near-natural landscape elements and protected areas within the urban area. Second, Vienna has an exceptional gradient in precipitation and temperature (Figure 5), which the bryophyte flora reflects. Oceanic influences from the west and Pannonian-continental influences from the east provide very different climatic conditions depending on the location within the city. With an average of 750 mm/year, the west of Vienna has twice as much precipitation as the east of Vienna (350 mm/year). Average temperatures are also approximately 2 °C lower in the west than in the east. Figure 5 also clearly shows an inner-city heat island, which is classic for cities [30,31]. Bryophyte diversity reflects this climatic diversity: for example, there are more Pannonian flora elements in the east and more sub-Atlantic flora elements in the west.

### 3.1. Comparison of Vienna’s Species Diversity with That of Other Cities

Studies on the species diversity of bryophytes in Austrian cities are available for Linz [32] and Salzburg [33]. For Linz, 319 taxa are known. For Salzburg, 323 taxa currently occur, and if we include all taxa mentioned at some time in history, the number of species is 444. In a sense, these species numbers are similar, even though the area size, the geological conditions, and especially the climate are very different in the three cities of Vienna, Linz, and Salzburg. The loss of species is much greater in Salzburg, with a decline of 27% compared to 14% in Vienna. This loss in species is undoubtedly related to the loss of sites rich in bryophytes (e.g., mires) in Salzburg or poorer historical recordings in Vienna. For the city of Graz, an overall survey is lacking, but a study on the areas of Schlossberg and the Botanical Garden [34] mentions 178 taxa. In a more recent study [35] on soil and rock bryophytes of the city, 70 taxa are listed.

Compared with other European cities, Vienna is thus in the top range: Berlin 385 taxa [36], Regensburg 230 taxa [37], Brussels 232 [38], Belgrade 210 taxa [39], Braunschweig 152 taxa [40], Cologne 143 taxa [39], Trento 136 taxa [41], Enna 80 taxa [42], Szczecin 73 taxa [43].

### 3.2. Implications for Management and Conservation

Forty-nine species historically known for Vienna were not found in the current surveys. Their loss might have several causes. At the time of the early bryologists, many sites were in villages and communities dominated by agriculture, which were incorporated into the city in the last hundred years (e.g., Roßau, Sievering, Stammersdorf, Floridsdorf). The disappearance of arable bryophytes such as *Anthoceros agrestis* or *Phaeoceros carolinianus* is probably due to this. Of course, urban sprawl is also partly to blame for the loss of some species. Wet meadows and fens have disappeared around Vienna. However, a significant impact on species number had the regulation of the Danube and the Wienfluss. On the banks of the unobstructed, markedly branched Danube and its “islands” (Figure 2) were diverse habitats, such as loamy, sandy, or gravelly floodplains. It is thus not surprising that species typical for the banks of large rivers have disappeared with these, such as 4 out of 5 *Ephemerum* species, *Bryum versicolor*, *Physcomitrium eurystomum*, or *P. sphaericum*.

Also, climate changes over the last 150 years reflect the loss of species that prefer higher humidity or cooler temperatures [44,45,46]. In Vienna, these include *Porella arboris-vitae*, *Ptilium crista-castrensis*, or the deadwood colonizer *Blepharostoma trichophyllum*. For other species, such as *Orthotrichum scanicum* or *Neckera pennata*, the influence of climate change is probably closely linked to that of air pollution [47,48,49].

**Figure 6:**
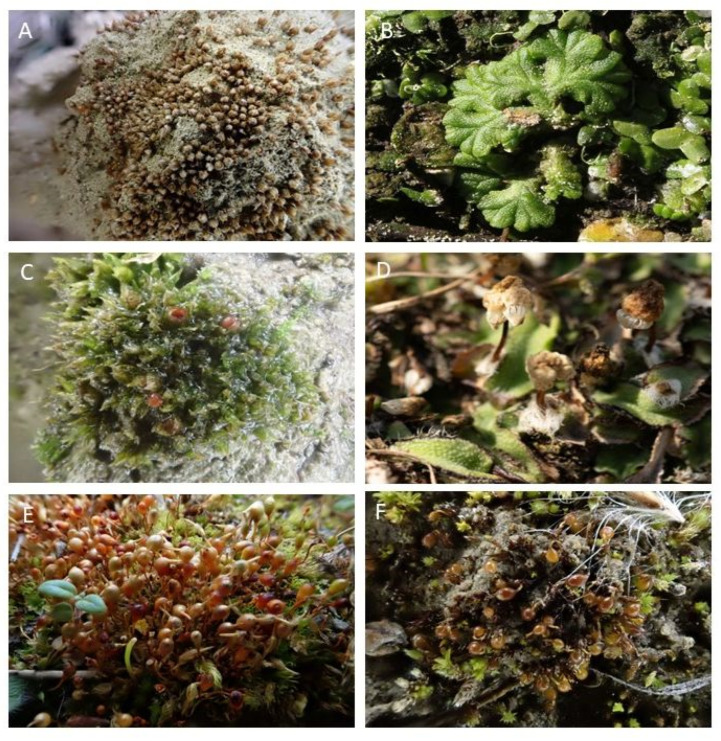
Rare species for which Vienna has a high responsibility: (**A**)—*Acaulon triquetrum*, (**B**)—*Ricciocarpos natans*, (**C**)—*Physcomitrium patens*, (**D**)—*Asterella saccata*, (**E**)—*Entosthodon fascicularis*, (**F**)—*Microbryum curvicollum*.

The species lost in Vienna have also become rare in the surrounding area. Of the 49 species that no longer occur in Vienna, 37 are classified as endangered in the Red List of Bryophytes of Lower Austria, 7 as extinct, and 12 as threatened with extinction (e.g., *Asterella saccata*, Figure 6D). Due to the site characteristics and size of the Donauinsel, the dry grasslands of the Lobau, and the habitats along streams in the Flyschwienerwald, some species are more frequent in Vienna than in neighboring Lower Austria concerning the area and marked with a “!” in Table A1 (Appendix A). These are *Acaulon triquetrum* (Figure 6A), *Aloina rigida*, *Cephaloziella stellulifera*, *Dicranella howei*, *Entosthodon fascicularis* (Figure 6E), *Microbryum curvicollum* (Figure 6F), *Physcomitrium patens* (Figure 6 C), *Pterygoneurum lamellatum*, *Ricciocarpos natans* (Figure 6B), *Seligeria pusilla*, *Blindiadelphus recurvatus*, and *Mesoptychia turbinata*. Vienna, therefore, is highly responsible for the conservation of these.

*Campylopus introflexus*, as the only neophytic bryophyte species in Vienna, occurred only regionally and in small populations.

A contribution to the protection of bryophytes is raising awareness that they are a significant part of the biodiversity of a large city and that the best way to protect them is to let them live where they emerge and grow on their own. A garden wall or roof surface overgrown with bryophytes is beautiful and increases biodiversity—not only of the bryophytes but also of microorganisms living in their protection—because each bryophyte cushion is a small microcosm [50]. Attempts to establish bryophytes outside their natural habitat are doomed to failure in Vienna for climatic reasons [51]. Attempting bryophytes for greening purposes on house walls and indoors should therefore be strictly rejected. Unfortunately, against better knowledge, the use of bryophytes in this area is in vogue. This use is associated with the destruction of bryophyte stands and the creatures hidden in them.

As the present data indicate, urban biodiversity is becoming increasingly important. Vienna’s diversity in bryophytes exceeds the species numbers of many cultural landscapes in rural areas [52,53]. Within cities, bryophytes are also indicators of a feel-good climate for people. The high number of boreo-temperate species in Vienna represents a cool–moderate climate that is also digestible for humans [54]. Bryophyte-free areas indicate areas within Vienna dominated by concrete and increasingly inhospitable for humans in the increasing heat and dry summer periods. Bryophyte-rich areas within the city can thus serve as guidelines for future urban planning [55]. In future studies, new bryophyte species will appear in Vienna. We have only recorded bryophytes from publicly accessible areas; further species might occur in old, structurally rich gardens in the suburbs and villa districts, which proved to be rich in species in other studies, e.g., [56].

## 4. Materials and Methods

For compiling the historical bryophyte flora, the studies of Zechmeister et al. [4,5] were primarily used. They are based on reports of Jacquin (1762, [6]), Welwitsch (1834, [7]), Garovaglio (1840, [8]), Pokorny (1854, [9]), Poetsch (1856, 1859, [10,11]), Reichardt (1858, [12]), Neilreich (1859, [13]), Juratzka (1882, [14]), Höfer (1887, [15]), Höhnel (1891, [16]), Heeg (1892, [17]), Matouschek (1905, [18]), and Onno (1941, [19]). Furthermore, we used site data of Baumgartner (1870–1955) from the archives and the herbarium of the Natural History Museum in Vienna (W), which have not been published so far.

We collected the current bryophyte data in the period between 1998 and 2023. We conducted no area-wide or quadrant-based search. In most cases, we investigated areas that were expected to have a rich bryophyte species assemblage or where we suspected rare species. Some target regions were determined by projects (e.g., on the dry grasslands of the Lobau or the Biosphärenpark Wienerwald, see funding) or were venues of the “Day of Biodiversity” or excursion destinations within the framework of courses held by the two authors at the University of Vienna. We also collected bryophytes during many walks and hikes in Vienna. In general, we recorded only bryophytes in publicly accessible areas.

Furthermore, we included data from diploma theses supervised by the first author [20,26,57,58,59] and from a previously published study of the authors [60].

The nomenclature follows Hodgetts et al. [61]. Endangerment classifications are according to the Red List of Bryophytes of Lower Austria [24]. Specimens of all species are in the private herbarium of H. G. Zechmeister.

The climate data originated from 15 climate stations operated by the municipality of Vienna and the Office of the Provincial Government of Lower Austria. The period of the climate data for the Kriging process covers the years 2014 to 2017. For modeling temperature and precipitation for the area of Vienna (Figure 5), we used the function “Ordinary Kriging” based on the model “spherical” implemented in the ArcGIS (Esri, version 10.8) program. We set the parameters “range”, “nugget”, “lag size”, “number of lags”, “search radius”, and the “number of neighbors” on default.

For the comparison of the biogeographic elements, we used a Chi-Square Test (*p* < 0.01) in the program SPSS (version 22.0).

## Data Availability

Not applicable.

## Appendix A

**Table A1:** List of species found in Vienna—nomenclature according to the Checklist of Bryophytes of Europe [61]; P: Phylum of bryophytes: A—Anthoceratophyta (hornworts), B—Bryophyta (mosses), M—Marchantiophyta (liverworts); O: occurrences: h—historical occurrence, n/h—current and historical occurrences, n—species for which there are no records in the historical literature. We found them during the current surveys, and they are published here for the first time; RL—Red List (according to the Red List of Bryophytes of Lower Austria [24]): R: !: responsibility of the city of Vienna for the respective bryophyte species; F—frequency: vr—very rare (1–2 current occurrences), r—rare (3–5 current occurrences), s—scattered (4–10 current occurrences), w—widespread (more than 10 current occurrences); locality: the locality is amended by information on the habitat, if available; when there are only historical reports of a species, we added the source.

Taxon	P	O	RL	R	F	Locality
*Abietinella abietina* (L. ex Hedw.) M.Fleisch. var. *abietina*	B	n/h	LC		s	Dry, sunny soils in the entire urban area.
*Acaulon muticum* (Hedw.) Müll.Hal.	B	n/h	EN		vr	Rooftop at the New General Hospital.
*Acaulon triquetrum* (Spruce) Müll.Hal.	B	n/h	VU	!	vr	Various dry grasslands in the Lobau.
*Alleniella besseri* (Lobarz.) S. Olsson, Enroth and D. Quandt	B	n	NT		r	Epiphyte in the Karbonatwienerwald.
*Alleniella complanata* (Hedw.) S. Olsson, Enroth and D. Quandt	B	n	LC		s	Epiphyte and on rocks at Vorderhainbach, Kasgraben, and Rodaun to Eichkogel.
*Aloina ambigua* (Bruch and Schimp.) Limpr.	B	n/h	EN		r	Dry grassland at Kreuzgrund, Lausgrund (Lobau), and Donauinsel.
*Aloina brevirostris* (Hook. and Grev.) Kindb.	B	h	RE			Danube at Stadlau [14].
*Aloina rigida* (Hedw.) Limpr.	B	n/h	VU	!	s	Dry grasslands “Kreuzgrund”, “Kontrollerwiese”, “Panozzalacke” (all Lobau).
*Amblystegium serpens* (Hedw.) Schimp.	B	n/h	LC		w	Soils and trees in the entire urban area.
*Anacamptodon splachnoides* (Froel. ex-Brid.) Brid.	B	h	CR			Neuwaldegg (Baumgartner, W, unpubl.).
*Anastrophyllum michauxii* (F. Weber) H. Buch	B	h	CR			Pötzleinsdorf [17].
*Aneura pinguis* (L.) Dumort.	M	n/h	LC		r	Soils in the Lainzer Tiergarten and Lobau.
*Anomodon viticulosus* (Hedw.) Hook. and Taylor	B	n/h	LC		s	Soils and rocks in the entire Wienerwald.
*Anthoceros agrestis* Paton	A	h	EN			Dornbach [7].
*Apopellia endiviifolia* (Dicks.) Nebel and D. Quandt	M	n/h	LC		s	Soils in the west of the town and Lobau.
*Archidium alternifolium* (Dicks. ex Hedw.) Mitt.	B	n/h	CR		vr	Banks of the oxbow "Panozzalacke" (Lobau).
*Asterella saccata* (Wahlenb.) A. Evans	M	n/h	CR		vr	Stone quarry at Rodaun.
*Atrichum angustatum* (Brid.) Bruch and Schimp.	B	n/h	LC		r	Soils at the Flyschwienerwald.
*Atrichum undulatum* (Hedw.) P. Beauv.	B	n/h	LC		w	Soils in the entire urban area.
*Barbilophozia barbata* (Schmidel ex Schreb.) Loeske	M	h	LC			Dornbach (Baumgartner, W., unpubl.).
*Barbula unguiculata* Hedw.	B	n/h	LC		w	Soils in the entire urban area.
*Bartramia ithyphylla* Brid.	B	h	NT			Neuwaldegg, Flyschwienerwald [9,14].
*Bartramia pomiformis* Hedw.	B	h	LC			Flyschwienerwald [14].
*Blasia pusilla* L.	M	n/h	LC		r	Soils at Mauerbach.
*Blepharostoma trichophyllum* (L.) Dumort. var. *trichophyllum*	M	h	NT			Dornbach [9].
*Blindiadelphus recurvatus* (Hedw.) Fedosov and Ignatov	B	n/h	LC	!	s	Rocks at Kolbeterberg and Latisberg.
*Brachytheciastrum velutinum* (L. ex Hedw.) Ignatov and Huttunen	B	n/h	LC		w	Soils in the entire urban area.
*Brachythecium albicans* (Neck. ex Hedw.) Schimp.	B	n/h	LC		s	Dry grassland at Panozzalacke (Lobau) and on various rooftops.
*Brachythecium campestre* (Müll.Hal.) Schimp.	B	n/h	NT		s	Various dry grasslands in the Lobau. Himmelswiese (Kalksburg), and on various rooftops.
*Brachythecium glareosum* (Bruch ex Spruce) Schimp. var. *glareosum*	B	n/h	LC		s	Various dry grasslands in the Lobau and on rooftops in the entire city.
*Brachythecium mildeanum* (Schimp.) Schimp.	B	n/h	EN		r	Wet meadows at Todtenwiese and in the Lobau.
*Brachythecium rivulare* Schimp.	B	n/h	LC		w	Along all water bodies.
*Brachythecium rutabulum* (L. ex Hedw.) Schimp.	B	n/h	LC		w	Soils in the entire urban area.
*Brachythecium salebrosum* (Hoffm. ex F. Weber and D. Mohr) Schimp.	B	n/h	LC		w	Soils in the entire urban area.
*Brachythecium tommasinii* (Sendtn. ex Boulay) Ignatov and Huttunen	B	n/h	LC		s	Rocks in the Lainzer Tiergarten and from Rodaun to Eichkogel.
*Bryoerythrophyllum recurvirostrum* (Hedw.) P.C. Chen	B	n/h	LC		r	Soils and rocks in the Lobau and at Steinhofgründe.
*Bryum algovicum* Sendtn. ex Müll.Hal.	B	n/h	LC		vr	Dry grassland at Kreuzgrund (Lobau).
*Bryum alpinum* Huds. ex With.	B	h	VU-R			Hermannskogel [14].
*Bryum archangelicum* Bruch and Schimp.	B	n	VU-R	!	s	Rooftops and various dry grasslands in the Lobau.
*Bryum argenteum* Hedw.	B	n/h	LC		w	Soils in the entire urban area.
*Bryum caespiticium* Hedw.	B	n/h	LC		w	Soils in the entire urban area.
*Bryum capillare* Hedw.	B	n/h	LC		w	Soils in the entire urban area.
*Bryum creberrimum* Taylor	B	n	LC		s	Dry grasslands in the Lobau and from Rodaun to Eichkogel.
*Bryum cyclophyllum* (Schwägr.) Bruch and Schimp.	B	h	RE			Floridsdorf, Prater (Baumgartner, W., unpubl.).
*Bryum dichotomum* Hedw.	B	n/h	LC		s	Riverbanks at the Donauinsel and in the Lobau.
*Bryum elegans* Nees var. *ferchelii*	B	n	LC		r	Soils at Himmelswiese (Kalksburg).
*Bryum intermedium* (Brid.) Blandow	B	n/h	DD		vr	Rooftop Old General Hospital.
*Bryum klinggraeffii* Schimp.	B	n	NT		s	Soils in the Lainzer Tiergarten and in Glasgraben.
*Bryum moravicum* Podp.	B	n/h	LC		w	Entire urban area.
*Bryum pallens* Sw. ex anon.	B	n/h	LC		r	Soils in the Prater.
*Bryum pallescens* Schleich. ex Schwägr.	B	n	LC		vr	Rooftop New General Hospital.
*Bryum pseudotriquetrum* (Hedw.) P. Gaertn., E. Mey. and Scherb. var. *pseudotriquetrum*	B	n/h	NT		vr	Wet soils at Kasgraben (Mauerbach).
*Bryum radiculosum* Brid.	B	n	VU		s	Soils at Kalksburg (Himmelwiese).
*Bryum rubens* Mitt.	B	n/h	LC		w	Soils in the entire urban area.
*Bryum turbinatum* (Hedw.) Turner	B	n/h	CR		vr	Banks of the Danube at Albern.
*Bryum uliginosum* (Brid.) Bruch and Schimp.	B	h	RE			Prater [9,14].
*Bryum versicolor* A. Braun ex Bruch and Schimp.	B	h	CR			Banks of the Danube at Floridsdorf, Kaisermühlen, Stadlau [14].
*Buxbaumia aphylla* Hedw.	B	h	VU			Dornbach [9,14].
*Calliergon cordifolium* (Hedw.) Kindb.	B	n	VU		vr	Fen at Aumüllerwiese (Lainzer Tiergarten).
*Calliergonella cuspidata* (L. ex Hedw.) Loeske	B	n/h	LC		s	Wet grasslands in the overall urban area.
*Calliergonella lindbergii* (Mitt.) Hedenäs	B	n	LC		s	Fen at Fasslwiese (Lainzer Tiergarten).
*Calypogeia azurea* Stotler and Crotz	M	n/h	LC		vr	Soils at Steinerne Lahn.
*Calypogeia fissa* (L.) Raddi	M	n	LC		s	Soils at Steinerne Lahn and Neuwaldegg.
*Calypogeia integristipula* Steph.	M	n	LC		r	Soils at Steinerne Lahn, Neuwaldegg, and Lainzer Tiergarten.
*Calypogeia muelleriana* (Schiffn.) Müll.Frib.	M	n	LC		r	Soils at Cobenzl.
*Campylium chrysophyllum* (Brid.) Lange	B	n/h	LC		s	Various dry grasslands in the Lobau, at Leopoldsberg and Hermannskogel.
*Campylium stellatum* (Schreb. ex Hedw.) Lange and C.E.O. Jensen	B	n/h	LC		r	Fen at Panozzalacke (Lobau).
*Campylopus introflexus* (Hedw.) Brid.	B	n	LC		r	Soils at Neuwaldegg and in the Sandsteinwienerwald.
Campylostelium saxicola (F. Weber and D. Mohr) Bruch and Schimp.	B	n/h	VU-R		r	Rocks at Latisberg, am Himmel and Steinerne Lahn.
*Cephalozia bicuspidata* (L.) Dumort.	M	n/h	LC		s	Soils at Ottakringer Wald, Cobenzl, and Gänshaufen (Lobau).
*Cephaloziella divaricata* (Sm.) Schiffn.	M	n/h	LC		r	Dry grassland at Mühlleiten (Lobau).
*Cephaloziella hampeana* (Nees) Schiffn.	M	n	VU		vr	Dry grassland “Gänshaufen” (Lobau).
*Cephaloziella rubella* (Nees) Warnst. var. *rubella*	M	n	LC		r	Various dry grasslands in the Lobau.
*Cephaloziella stellulifera* (Taylor ex Spruce) Schiffn.	M	n/h	RE	!	r	Dry grassland “Kontrollerwiese” (Lobau).
*Ceratodon conicus* (Hampe) Lindb.	B	n	VU		vr	Rooftop New General Hospital.
*Ceratodon purpureus* (Hedw.) Brid.	B	n/h	LC		w	Soils in the entire urban area.
*Chiloscyphus pallescens* (Ehrh. ex Hoffm.) Dumort.	M	n/h	LC		s	Wet soils at the Lainzer Tiergarten.
*Chiloscyphus polyanthos* (L.) Corda	M	n/h	LC		s	Banks of various oxbows in the Lobau.
*Cinclidotus danubicus* Schiffn. and Baumgartner	B	h	CR			Taborbrücke (Baumgartner, W., unpubl.).
*Cinclidotus fontinaloides* (Hedw.) P. Beauv.	B	n/h	LC		w	Danube, Donaukanal.
*Cinclidotus riparius* (Host ex Brid.) Arn.	B	n/h	LC		w	Danube, Donaukanal.
*Cirriphyllum crassinervium* (Taylor) Loeske and M. Fleisch.	B	n/h	LC		r	Rocks from Rodaun to Eichkogel.
*Cirriphyllum piliferum* (Schreb. ex Hedw.) Grout	B	n/h	LC		w	Soils in the entire urban area.
*Climacium dendroides* (Hedw.) F. Weber and D. Mohr	B	n/h	LC		w	Soils in the entire urban area.
*Conocephalum conicum* (L.) Dumort.	M	n/h	LC		s	Wet soils at Halterbach (Mauerbach) and Lainzer Tiergarten.
*Cratoneuron filicinum* (L. ex Hedw.) Spruce	B	n/h	LC		s	Water bodies in the entire urban area.
*Ctenidium molluscum* (Hedw.) Mitt. var. *molluscum*	B	n/h	LC		s	Soils and rocks at Cobenzl, from Rodaun to Eichkogel and Lainzer Tiergarten.
*Dichodontium pellucidum* (Hedw.) Schimp.	B	n/h	LC		r	Soils at Neuwaldegg.
*Dicranella heteromalla* (Hedw.) Schimp.	B	n/h	LC		w	Soils in the entire Flyschwienerwald.
*Dicranella howei* Renauld and Cardot	B	n	VU	!	r	Dry grassland “Kreuzgrund” (Lobau) and Central Cemetery.
*Dicranella rufescens* (Dicks.) Schimp.	B	n/h	VU-R		r	Soils at Steinerne Lahn.
*Dicranella schreberiana* (Hedw.) Dixon	B	n/h	LC		s	Soils in the entire Flyschwienerwald.
*Dicranella varia* (Hedw.) Schimp.	B	n/h	LC		w	Soils in the entire Flyschwienerwald and Lobau.
*Dicranodontium denudatum* (Brid.) E. Britton	B	n	LC		vr	Deadwood at Johannser Kogel (Lainzer Tiergarten).
*Dicranum bonjeanii* De Not.	B	h	VU			Neuwaldegg, Haltertal, Muschinger Wiese (Baumgartner, W., unpubl.).
*Dicranum fuscescens* Sm.	B	n	LC		vr	Deadwood at Johannser Kogel (Lainzer Tiergarten).
*Dicranum montanum* Hedw.	B	n/h	LC		w	Soils in the entire Flyschwienerwald and Lobau.
*Dicranum polysetum* Sw. ex anon.	B	n/h	LC		s	Soils at Heuberg (Neuwaldegg), Steinerne Lahn, and surroundings.
*Dicranum scoparium* Hedw.	B	n/h	LC		w	Soils in the entire Flyschwienerwald and Lobau.
*Dicranum tauricum* Sapjegin	B	n	LC		r	Epiphyte at Johannser Kogel (Lainzer Tiergarten).
*Dicranum undulatum* Schrad. ex Brid.	B	h				Knödelhütte bei Hütteldorf (Baumgartner, W., unpubl.).
*Didymodon acutus* (Brid.) K. Saito	B	n/h	LC		r	Rooftops, dry grasslands “Kreuzgrund” and “Lausgrundwasser” (Lobau).
*Didymodon cordatus* Jur.	B	n	NT		r	Various dry grasslands at Leopoldsberg, Lobau, Himmelwiese.
*Didymodon fallax* (Hedw.) R.H. Zander	B	n/h	LC		s	Soils in the entire urban area.
*Didymodon ferrugineus* (Schimp. ex Besch.) M.O. Hill	B	n/h	LC		s	Soils in the entire urban area.
*Didymodon rigidulus* Hedw.	B	n/h	LC		r	Soil at the Maurer Wald.
*Didymodon spadiceus* (Mitt.) Limpr.	B	n/h	LC		s	Water bodies in the Wienerwald.
*Didymodon tophaceus* (Brid.) Lisa	B	n/h	NT		s	Water bodies in the Wienerwald.
*Didymodon vinealis* (Brid.) R.H. Zander	B	n/h	EN		r	Dry grasslands “Kreuzgrund” and “Kontrollerwiese” (both Lobau), “Latisberg”.
*Diphyscium foliosum* (Hedw.) D.Mohr	B	n/h	LC		s	Soils in the entire Flyschwienerwald.
*Diplophyllum albicans* (L.) Dumort.	M	n/h	LC		r	Soils at Steinerne Lahn.
*Diplophyllum obtusifolium* (Hook.) Dumort.	M	n/h	LC		r	Soils at Steinerne Lahn.
*Ditrichum heteromallum* (Hedw.) E. Britton	B	n/h	LC		w	Soils in the entire Flyschwienerwald.
*Ditrichum pallidum* (Hedw.) Hampe	B	n/h	VU-R		r	Soils at Steinerne Lahn.
*Ditrichum pusillum* (Hedw.) Hampe	B	n/h	NT		r	Soils at Ottakringer Wald.
*Drepanocladus aduncus* (Hedw.) Warnst.	B	n/h	LC		s	Various oxbows in the Lobau and in the Mauthner- and Krebsenwasser (both Prater).
*Drepanocladus lycopodioides* (Brid.) Warnst.	B	h	CR			Eckbach bei Dornbach (Baumgartner, W., upubl.).
*Drepanocladus polygamus* (Schimp.) Hedenäs	B	n/h	EN		r	Fen at Mühlwasser (Lobau).
*Drepanocladus sendtneri* (Schimp. ex H. Müll.) Warnst.	B	h	CR			Oxbow “Heustadlwasser” (Prater) (Baumgartner, W., upubl.).
*Encalypta streptocarpa* Hedw.	B	n/h	LC		w	Soils in the entire urban area.
*Encalypta vulgaris* Hedw.	B	n/h	LC		s	Dry grasslands in the overall urban area.
*Entodon concinnus* (De Not.) Paris	B	n/h	LC		s	Dry grasslands in the entire urban area.
*Entodon schleicheri* (Schimp.) Demet.	B	n	CR		r	Sunny sites at Latisberg and Leopoldsberg.
*Entosthodon fascicularis* (Hedw.) Müll.Hal.	B	n/h	CR	!	r	Rooftops New General Hospital, dry grassland at Mühlleiten (Lobau), and the Botanical Garden.
*Entosthodon muhlenbergii* (Turner) Fife	B	h	CR			Glacis, Schönbrunn [7,14].
*Ephemerum cohaerens* (Hedw.) Hampe	B	h	CR			Bank of the Danube [9,14].
*Ephemerum recurvifolium* (Dicks.) Boulay	B	h	CR			Botanical garden, Dornbach [7,9].
*Ephemerum serratum* (Scheb. ex Hedw.) Hampe	B	n/h	VU		r	Bank of the Panozzalacke (Lobau), and at Gütenbachtal.
*Ephemerum sessile* (Bruch) Müll.Hal.	B	h	CR			Belvedere [7].
*Eucladium verticillatum* (With.) Bruch and Schimp.	B	n/h	NT		vr	Tufa-spring at Kaltbründlwiese (Lainzer Tiergarten).
*Eurhynchiastrum pulchellum* var. *praecox* (Hedw.) Ochyra and Żarnowiec	B	n/h	LC		vr	Soils at Johannser Kogel (Lainzer Tiergarten) and Moosgraben.
*Eurhynchium angustirete* (Broth.) T.J. Kop.	B	n/h	LC		s	Soils in the entire urban area.
*Exsertotheca crispa* (Hedw.) S. Olsson, Enroth and D. Quandt	B	n	LC		s	Trees and rocks at Vorderhainbach, Kasgraben, and from Rodaun to Eichkogel.
*Fissidens adianthoides* Hedw.	B	n/h	NT		r	Fen at Kaltbründlwiese (Lainzer Tiergarten) and at Pfaffenberg.
*Fissidens bryoides* Hedw.	B	n/h	LC		w	Soils in the entire Wienerwald and in the Lobau.
*Fissidens crassipes* Wilson ex Bruch and Schimp.	B	n/h	NT		r	Banks of the Donaukanal.
*Fissidens dubius* P. Beauv.	B	n/h	LC		s	Soils in the entire Karbonatwienerwald and Am Himmel.
*Fissidens exilis* Hedw.	B	n/h	VU-R		s	Soils in the entire urban area.
*Fissidens incurvus* Starke ex Röhl.	B	h	DD			Flyschwienerwald, Sievering, Hermannskogel ([14], Baumgartner, W., upubl.).
*Fissidens pusillus* (Wilson) Milde	B	n/h	LC		s	Along water bodies at Sievering, Pfaffenberg, Cobenzl, and surroundings, Neuwaldegg.
*Fissidens taxifolius* Hedw. subsp. *taxifolius*	B	n/h	LC		s	Soils in the entire urban area.
*Fissidens viridulus* (Sw. ex anon.) Wahlenb.	B	h				Schönbrunn [7].
*Flexitrichum flexicaule* (Schwägr.) Ignatov and Fedosov	B	n/h	LC		s	Various dry grasslands in the Lobau and elsewhere.
*Fontinalis antipyretica* L. ex Hedw.	B	n/h	LC		w	Donaukanal, water bodies in the Wienerwald and Lobau.
*Fontinalis hypnoides* C. Hartm.	B	h	CR			Oxbow at Heustadlwasser and Freudenau (Prater).
*Fossombronia pusilla* (L.) Nees	M	h	DD			Neuwaldegg.
*Fossombronia wondraczekii* (Corda) Lindb.	M	n/h	NT		r	Soils at Fasslwiese (Lainzer Tiergarten).
*Frullania dilatata* (L.) Dumort.	M	n/h	LC		w	Epiphyte in the entire urban area outside the city center.
*Frullania tamarisci* (L.) Dumort.	M	n/h	VU		r	Epiphyte at Gütenbachtal.
*Funaria hygrometrica* Hedw.	B	n/h	LC		w	Soils in the entire urban area.
*Fuscocephaloziopsis lunulifolia* (Dumort.) Váňa and L. Söderstr.	M	n	LC		vr	Soils at Ottakringer Wald.
*Grimmia anodon* Bruch and Schimp.	B	n	LC		vr	Sunny rock at Himmelswiese (Kalksburg).
*Grimmia orbicularis* Bruch ex Wilson	B	n	LC		r	Sunny rocks at Leopoldsberg and Himmelwiese (Kalksburg).
*Grimmia pulvinata* (Timm. ex Hedw.) Sm.	B	n/h	LC		w	Rocks and buildings in the entire urban area.
*Grimmia tergestina* Tomm. ex Bruch and Schimp.	B	n	LC		vr	Sunny rocks at Rodaun.
*Gymnostomum aeruginosum* Sm.	B	n/h	LC		vr	Wet shady rock at Fasslwiese (Lainzer Tiergarten).
*Hedwigia ciliata* (Hedw.) P. Beauv. var. *ciliata*	B	n	LC		r	Epiphyte (!) at Cobenzl/Latisberg.
*Hennediella heimii* (Hedw.) R.H. Zander	B	h	RE			Erdberg [14].
*Herzogiella seligeri* (Brid.) Z. Iwats.	B	n/h	LC		vr	Deadwood at Satzberg.
*Heterocladiella dimorpha* (Brid.) Ignatov and Fedosov	B	h	LC			Soils and roots at Dornbach, Neuwaldegg, Kahlenberg, and Hütteldorf.
*Homalia trichomanoides* (Hedw.) Brid.	B	n/h	LC		s	Epiphyte at Satzberg and in the Lobau.
*Homalothecium lutescens* (Hedw.) H. Rob.	B	n/h	LC		w	Dry grasslands and rooftops in the entire urban area.
*Homalothecium philippeanum* (Spruce) Schimp.	B	n/h	LC		s	Soils from Rodaun to Eichkogel.
*Homalothecium sericeum* (Hedw.) Schimp.	B	n/h	LC		s	Soils and rocks in the entire urban area.
*Homomallium incurvatum* (Schrad. ex Brid.) Loeske	B	n/h	LC		s	Rocks at the Central Cemetery, Schwarzenbergpark and Lainzer Tiergarten.
*Hygroamblystegium fluviatile* (Hedw.) Loeske	B	n/h	NT		s	Oxbow “Panozzalacke” (Lobau) and in the Danube.
*Hygroamblystegium humile* (P. Beauv.) Vanderp., Goffinet and Hedenäs	B	n/h	EN		vr	Bank of the oxbow Panozzalacke (Lobau).
*Hygroamblystegium varium* (Hedw.) Mönk.	B	n/h	LC		w	Soils in the entire urban area.
*Hygrohypnum luridum* (Hedw.) Jenn.	B	n/h	LC		r	Water bodies at Glasgraben and Eichwiese, Danube.
*Hylocomiadelphus triquetrus* (Hedw.) Ochyra and Stebel	B	n/h	LC		s	Soils in the Lobau, Central Cemetery, and Western Wienerwald.
*Hylocomium splendens* (Hedw.) Schimp.	B	n/h	LC		s	Soils at Lainzer Tiergarten, Lobau and Halterbach.
*Hymenostylium recurvirostrum* (Hedw.) Dixon	B	n/h	LC		vr	Wet rock at Kaltbründlwiese (Lainzer Tiergarten).
*Hymenostylium xerophilum* Köckinger and Kucera	B	n	DD		vr	Sunny rock at Lainzer Tiergarten.
*Hypnum andoi* A.J.E. Sm.	B	n	LC		vr	Epiphyte at Steinerne Lahn.
*Hypnum cupressiforme* Hedw. var. *cupressiforme*	B	n/h	LC		w	Entire urban area.
*Hypnum cupressiforme* var. *lacunosum* Brid.	B	n/h	LC		s	Dry grasslands in the entire urban area.
*Isothecium alopecuroides* (Lam. ex Dubois) Isov.	B	n/h	LC		w	Entire Flyschwienerwald.
*Jungermannia atrovirens* Dumort.	M	n/h	LC		r	Wet rock at Latisberg.
*Kandaea elodes* (Lindb.) Jan Kučera and Hedenäs	B	h	CR			Prater, Neuwaldegg, Haltertal (Baumgartner, W., upubl.).
*Kindbergia praelonga* (L. ex Hedw.) Ochyra	B	n/h	LC		vr	Floodplain forest in the Lobau.
*Lejeunea cavifolia* (Ehrh.) Lindb.	M	n/h	LC		r	Epiphyte at Mauerbach.
*Lepidozia reptans* (L.) Dumort.	M	n/h	LC		s	Deadwood at Halterbachtal.
*Leptobryum pyriforme* (Hedw.) Wilson	B	n/h	LC		s	Soils in the Botanical Garden.
*Leptodictyum riparium* (Hedw.) Warnst.	B	n/h	LC		r	Danube, Donaukanal.
*Leskea polycarpa* Ehrh. ex Hedw.	B	n/h	LC			Epiphyte in the Lobau, Donauinsel, and Prater.
*Leucobryum glaucum* (Hedw.) Ångstr.	B	n/h	LC		s	Soils at the Johannserkogel (Lainzer Tiergarten), Pfaffenberg, Cobenzl, and surroundings.
*Leucobryum juniperoideum* (Brid.) Müll.Hal.	B	n	LC		s	Soils at Steinerne Lahn, Cobenzl, and surroundings.
*Leucodon sciuroides* (Hedw.) Schwägr.	B	n/h	LC		w	Epiphyte in the entire urban area outside the city center.
*Lewinskya affinis* (Schrad. ex Brid.) F. Lara, Garilleti and Goffinet	B	n/h	LC		s	Epiphyte in the Wienerwald, Central Cemetery, and Lobau.
*Lewinskya rupestris* (Schleich. ex Schwägr.) F. Lara, Garilleti and Goffinet	B	n/h	NT		r	Rocks at Heuberg and Wildgrube.
*Lewinskya speciosa* (Nees) F. Lara, Garilleti and Goffinet	B	n	LC		s	Epiphyte in the Wienerwald, Central Cemetery and Lobau.
*Lewinskya striata* (Hedw.) F. Lara, Garilleti and Goffinet	B	n/h	LC		s	Epiphyte in the Wienerwald and Central Cemetery.
*Lophocolea bidentata* (L.) Dumort.	M	n/h	LC		s	Deadwood at Ottakringer Wald and Satzberg.
*Lophocolea heterophylla* (Schrad.) Dumort.	M	n/h	LC		s	Deadwood at Steinerne Lahn, Wildgrube, and Lobau.
*Lophocolea minor* Nees	M	n/h	LC		r	Soils at Lobau and Kalksburg.
*Lophoziopsis excisa* (Dicks.) Konstant. and Vilnet	M	h	LC			Salmannsdorf [17].
*Lunularia cruciata* (L.) Dumort. ex Lindb.	M	n/h	LC		s	Lobau, Donaukanal, parks.
*Mannia fragrans* (Balbis) Frye and L. Clark	M	n/h	VU		vr	Soils at Botanical Garden and in some city areas.
*Marchantia polymorpha* L. subsp. *polymorpha*	M	n/h	LC		s	Banks at the Donaukanal.
*Marchantia polymorpha* subsp. *ruderalis* Bischl. and Boisselier	M	n/h	VU-R		w	Soils in the entire urban area.
*Marchantia quadrata* Scop.	M	n	LC		r	Rocks from Rodaun to Eichkogel.
*Marsupella funckii* (F. Weber and D. Mohr) Dumort.	M	h	VU			Neuwaldegg, Hermannskogel [17].
*Mesoptychia collaris* (Nees) L. Söderstr. and Váňa	M	n/h	LC		r	Soils at Michaelerberg and Schwarzenbergpark.
*Mesoptychia turbinata* (Raddi) L. Söderstr. and Váňa	M	n	VU-R	!	vr	Dry grassland “Kreuzgrund” (Lobau).
*Metzgeria conjugata* Lindb.	M	n/h	LC		s	Rocks and trees in the Flyschwienerwald.
*Metzgeria furcata* (L.) Dumort.	M	n/h	LC		s	Epiphyte in the entire Wienerwald.
*Microbryum curvicollum* (Ehrh. ex Hedw.) R.H. Zander	B	n/h	VU	!	r	Various dry grasslands in the Lobau, Donauinsel.
*Microbryum davallianum* (Sm.) R.H. Zander	B	n/h	EN		r	Dry grasslands Kreuzgrund and Kontrolerwiese (Lobau), Donauinsel.
*Microbryum floerkeanum* (F. Weber and D. Mohr) Schimp.	B	n/h	EN		vr	Dry grassland Fuchshäufl (Lobau).
*Microbryum starckeanum* (Hedw.) R.H. Zander	B	n	EN		vr	Dry grassland "Kreuzgrund" (Lobau).
*Mnium hornum* Hedw.	B	n	LC		s	Soils in the entire Wienerwald.
*Mnium marginatum* (Dicks.) P. Beauv.	B	n/h	LC		s	Soils in the entire Wienerwald.
*Mnium spinosum* (Voit) Schwägr.	B	n/h	LC		s	Soils at Pfaffenberg.
*Mnium stellare* Reichard ex Hedw.	B	n/h	LC		s	Soils at Vorderhainbach and Kasgraben.
*Nardia scalaris* Gray	M	n/h	LC		r	Soils at Satzberg and Ottakringer Wald.
*Neckera pennata* Hedw.	B	h	CR			Dornbach (Baumgartner, W., upubl., [7]).
*Nogopterium gracile* (Hedw.) Crosby and W.R. Buck	B	n	DD		vr	Soil at Belvedere.
*Nowellia curvifolia* (Dicks.) Mitt.	M	n/h	LC		r	Deadwood at Gänshaufen (Lobau).
*Nyholmiella obtusifolia* (Brid.) Holmen and E. Warncke	B	n/h	LC		s	Epiphyte in the entire Wienerwald, Lobau, and Central Cemetery.
*Orthotrichum anomalum* Hedw.	B	n/h	LC		w	Rocks and walls in the entire urban area.
*Orthotrichum diaphanum* Schrad. ex Brid.	B	n/h	LC		w	Epiphyte in the entire urban area.
*Orthotrichum pallens* Bruch ex Brid.	B	n/h	LC		s	Epiphyte in the entire Wienerwald, Central Cemetery, and Lobau.
*Orthotrichum patens* Bruch ex Brid.	B	n	LC		r	Epipyhyte in the Lainzer Tiergarten.
*Orthotrichum pumilum* Sw. ex anon.	B	n/h	LC		w	Epiphyte in the entire urban area.
*Orthotrichum scanicum* Grönvall	B	h	RE			Neuwaldegg, Hermannskogel, hameau [14].
*Orthotrichum schimperi* Hammar	B	n	DD		s	Epiphyte in the entire Wienerwald, Central Cemetery, and Lobau.
*Orthotrichum stramineum* Hornsch. ex Brid.	B	h	LC			Neuwaldegg, Dornbach [9].
*Orthotrichum tenellum* Bruch ex Brid.	B	h	CR			Between Pötzleinsdorf and Neuwaldegg [9].
*Oxyrrhynchium hians* (Hedw.) Loeske var. *hians*	B	n/h	LC		w	Soils in the entire urban area.
*Oxyrrhynchium schleicheri* (R. Hedw.) Röll	B	n/h	LC		r	Rooftops at the General Hospital and soils at the Central Cemetery.
*Oxyrrhynchium speciosum* (Brid.) Warnst.	B	n/h	NT		r	Banks of the oxbow in the Lobau.
*Palustriella commutata* (Hedw.) Ochyra var. *commutata*	B	n/h	LC		r	Water springs at Kasgraben (Mauerbach).
*Pedinophyllum interruptum* (Nees) Kaal.	M	n/h	LC		r	Soils at Dornbach.
*Pellia epiphylla* (L.) Corda	M	n/h	LC		s	Soils in the west of the town and Lobau.
*Phaeoceros carolinianus* (Michx.) Prosk.	A	h	CR			Dornbach (Baumgartner, W., unpubl., [9]).
*Philonotis calcarea* (Bruch and Schimp.) Schimp.	B	h	LC			Hainbach [14].
*Philonotis fontana* (L. ex Hedw.) Brid.	B	n/h	NT		r	Water spring at Kasgraben (Mauerbach) and Neuwaldegg.
*Philonotis marchica* (Hedw.) Brid.	B	n	DD		r	City center.
*Physcomitrium eurystomum* Sendtn.	B	h	VU			Bank of the Danube [17].
*Physcomitrium patens* (Hedw.) Mitt.	B	n	VU		s	Banks of the entire Donauinsel and Lobau.
*Physcomitrium pyriforme* (Hedw.) Bruch and Schimp.	B	n/h	LC		s	Banks of the oxbows in the Lobau, Gütenbachtal.
*Physcomitrium sphaericum* (C.F. Ludw. ex Schkuhr) Brid.	B	h	RE			Bank of the Danube at Kagran, Floridsdorf, Haltertal [14,17].
*Plagiochila asplenioides* (L. emend. Taylor) Dumort.	M	n/h	LC		w	Soils at Gütenbachtal, in the Lainzer, Tiergarten, and Flyschwienerwald.
*Plagiochila porelloides* (Torr. ex Nees) Lindenb.	M	n	LC		s	Soils in the Flyschwienerwald.
*Plagiomnium affine* (Blandow ex Funck) T.J. Kop.	B	n/h	LC		s	Soils at Latisberg and Gütenbachtal.
*Plagiomnium cuspidatum* (Hedw.) T.J. Kop.	B	n/h	LC		w	Soils in the entire urban area.
*Plagiomnium elatum* (Bruch and Schimp.) T.J. Kop.	B	n	LC		s	Soils in the Lobau and at Prater.
*Plagiomnium ellipticum* (Brid.) T.J. Kop.	B	n	EN		r	Wet soil at Kasgraben (Mauerbach).
*Plagiomnium rostratum* (Schrad.) T.J. Kop.	B	n/h	LC		s	Soil at the Wienerwald and Lobau.
*Plagiomnium undulatum* (Hedw.) T.J. Kop.	B	n/h	LC		w	Soils in the entire urban area.
*Plagiothecium cavifolium* (Brid.) Z. Iwats.	B	n/h	LC		s	Soils at Steinhofgründe and Sandsteinwienerwald.
*Plagiothecium denticulatum* (L. ex Hedw.) Schimp.	B	n/h	LC		s	Soils in the Flyschwienerwald.
*Plagiothecium laetum* Schimp. var. *laetum*	B	n/h	LC		s	Soils in the Sandsteinwienerwald.
*Plagiothecium nemorale* (Mitt.) A. Jaeger	B	n/h	LC		s	Soils in the Flyschwienerwald.
*Plasteurhynchium striatulum* (Spruce) M. Fleisch.	B	n/h	LC		r	Rocks at the Maurer Wald.
*Platygyrium repens* (Brid.) Schimp.	B	n	LC		w	Epiphyte in the entire urban area.
*Pleuridium acuminatum* Lindb.	B	n/h	VU		r	Dry grassland at Fuchshäufl (Lobau).
*Pleuridium subulatum* (Hedw.) Rabenh.	B	n/h	NT		r	Soils at Central Cemetery and Leopoldsberg.
*Pleurozium schreberi* (Willd. ex Brid.) Mitt.	B	n/h	LC		s	Soils in the Sandsteinwienerwald.
*Pogonatum aloides* (Hedw.) P. Beauv.	B	n/h	LC		s	Soils in the Flyschwienerwald.
*Pogonatum nanum* (Schreb. ex Hedw.) P. Beauv.	B	n/h	CR		r	Soils at Satzberg and Latisberg.
*Pogonatum urnigerum* (L. ex Hedw.) P. Beauv.	B	n/h	LC		s	Soils at Halterbachtal.
*Pohlia cruda* (L. ex Hedw.) Lindb.	B	n/h	LC		s	Soils in the Flyschwienerwald,
*Pohlia elongata* Hedw. subsp. *elongata*	B	h	VU-R			Hameau [14].
*Pohlia melanodon* (Brid.) A.J. Shaw	B	n/h	LC		s	Soils in the Flyschwienerwald.
*Pohlia nutans* (Hedw.) Lindb. subsp. *nutans*	B	n/h	LC		s	Soils in the Flyschwienerwald.
*Pohlia wahlenbergii* (F. Weber and D. Mohr) A.L. Andrews var. *wahlenbergii*	B	n/h	LC		vr	Water spring at Kaltbründlwiese.
*Polytrichum commune* Hedw.	B	n/h	LC		s	Soils in the Flyschwienerwald.
*Polytrichum formosum* Hedw.	B	n/h	LC		w	Soils in the Flyschwienerwald.
*Polytrichum juniperinum* Willd. ex Hedw.	B	n/h	LC		r	Soils at Neuwaldegg.
*Polytrichum perigoniale* Michx.	B	n/h	LC		s	Soils in the Flyschwienerwald.
*Polytrichum piliferum* Schreb. ex Hedw.	B	n/h	LC		vr	Soils at Steinerne Lahn.
*Porella arboris-vitae* (With.) Grolle	M	h	NT			Erdberg [17].
*Porella platyphylla* (L.) Pfeiff.	B	n/h	LC		w	Epiphyte in the entire urban area outside the city center.
*Pottia truncata* (Hedw.) Bruch and Schimp.	B	n/h	LC		s	Soils at Steinhofgründe, Rodaun, and Donauinsel.
*Pseudanomodon attenuatus* (Hedw.) Ignatov and Fedosov	B	n/h	LC		w	Epiphyte and on rocks in the entire urban area outside the city center.
*Pseudephemerum nitidum* (Hedw.) Loeske	B	n/h	EN		vr	Banks of the Donauinsel.
*Pseudoamblystegium subtile* (Hedw.) Vanderp. and Hedenäs	B	n/h	LC		s	Trees and roots in the entire Flyschwienerwald.
*Pseudocampylium radicale* (P. Beauv.) Vanderp. and Hedenäs	B	n	VU		vr	Banks of the Panozzalacke (Lobau).
*Pseudocrossidium hornschuchianum* (Schultz) R.H. Zander	B	n/h	LC		s	Various dry grasslands in the Lobau and on rooftops.
*Pseudocrossidium revolutum* (Brid.) R.H. Zander	B	n/h	EN		r	Rooftops in the entire area.
*Pseudoleskeella catenulata* (Brid. ex Schrad.) Kindb.	B	n/h	LC		s	Rocks at the Karbonatwienerwald and Leopoldsberg.
*Pseudoleskeella nervosa* (Brid.) Nyholm	B	n/h	LC		r	Rocks at Leopoldsberg and Lainzer Tiergarten.
*Pseudoscleropodium purum* (L. ex Hedw.) M.Fleisch.	B	n/h	LC		s	Soils in the entire urban area.
*Pseudotaxiphyllum elegans* (Brid.) Z. Iwats.	B	n/h	LC		r	Soils at Steinhofgründe and Steinerne Lahn.
*Pterigynandrum filiforme* Hedw. var. *filiforme*	B	n/h	LC		w	Epiphyte in the Flyschwienerwald and Lobau.
*Pterygoneurum lamellatum* (Lindb.) Jur.	B	n/h	EN	!	vr	Sunny, dry sites at the Donauinsel and Leopoldsberg.
*Pterygoneurum ovatum* (Hedw.) Dixon	B	n/h	VU		s	Dry grassland Kreuzgrund (Lobau), soils at Donauinsel, Central Cemetery, and Leopoldsberg.
*Pterygoneurum subsessile* (Brid.) Jur.	B	n/h	EN		r	Dry grassland Fuchshäufl (Lobau),
*Ptilium crista-castrensis* (L. ex Hedw.) De Not.	B	h	NT			Dornbach [7,14].
*Pylaisia polyantha* (Hedw.) Schimp.	B	n/h	LC		s	Epiphyte in Western districts of Vienna.
*Racomitrium canescens* (Timm. ex Hedw.) Brid. subsp. *canescens*	B	n/h	LC		r	Dry grassland “Panozzalacke” (Lobau).
*Racomitrium ericoides* (Brid.) Brid.	B	h	VU-R			Flyschwienerwald, Laaerberg, Neuwaldegg, Mauer, Hermannskogel [14].
*Radula complanata* (L.) Dumort.	M	n/h	LC		w	Epiphyte in the entire urban area outside the city center.
*Radula lindenbergiana* Gottsche ex C. Hartm.	M	n/h	VU		r	Epiphyte Johannser Kogel (Lainzer Tiergarten).
*Rhizomnium punctatum* (Hedw.) T.J. Kop.	B	n/h	LC		s	Deadwoods and along water bodies in the Wienerwald, soils at General Hospital rooftops.
*Rhodobryum roseum* (Hedw.) Limpr.	B	n/h	LC		s	Soils at Steinhofgründe and in the Lobau.
*Rhynchostegiella jacquinii* (Garov.) Limpr.	B	n/h	VU-R		vr	Rocks at water bodies at Kolbeterberg.
*Rhynchostegiella tenella* (Dicks.) Limpr.	B	n	LC		vr	Rock at Johannser Kogel (Lainzer Tiergarten).
*Rhynchostegium confertum* (Dicks.) Schimp.	B	n/h	VU-R		r	Rocks and roots at Steinhofgründe, Pfaffenberg, and Lainzer Tiergarten.
*Rhynchostegium megapolitanum* (Blandow ex F. Weber and D. Mohr) Schimp.	B	n/h	LC		r	Rooftop at the New General Hospital.
*Rhynchostegium murale* (Neck. ex Hedw.) Schimp.	B	n/h	LC		s	Rocks in the entire area.
*Rhynchostegium riparioides* (Hedw.) Cardot	B	n/h	LC		s	In the Danube.
*Rhynchostegium rotundifolium* (Scop. ex Brid.) Schimp.	B	n/h	VU-R		r	Rocks at the Central Cemetery and at Neuwaldegg.
*Rhytidiadelphus squarrosus* (L. ex Hedw.) Warnst.	B	n/h	LC		w	Soils in the entire urban area.
*Rhytidium rugosum* (Ehrh. ex Hedw.) Kindb.	B	n/h	LC		s	Dry grasslands in the entire urban area.
*Riccardia multifida* (L.) Gray	M	h	VU			Stadlau [62].
*Riccia bifurca* Hoffm.	M	n/h	EN		r	Bank of the oxbow Mühlwasser (Lobau).
*Riccia cavernosa* Hoffm.	M	n/h	VU		vr	Bank of the oxbow Mühlwasser (Lobau),
*Riccia ciliata* Hoffm.	M	h	EN			Oberlaa [7].
*Riccia fluitans* L.	M	n/h	NT		s	Oxbows "Mauthner- and Krebsenwasser" (Prater) and various oxbows in the Lobau,
*Riccia frostii* Austin	M	h	RE			Wienfluss Zollamt [9].
*Riccia glauca* L.	M	n/h	NT		s	Banks of the oxbows of Mauthner- and Krebsenwasser (Prater) and at the Donauinsel.
*Riccia sorocarpa* Bisch.	M	n/h	EN		vr	Bank of the oxbow “Mühlwasser” (Lobau).
*Riccia warnstorfii* Limpr. ex Warnst.	M	n	CR		r	Banks of the oxbow “Mauthnerwasser” (Prater).
*Ricciocarpos natans* (L.) Corda	M	n/h	EN	!	r	Banks of the oxbows “Mauthnerwasser” (Prater) and Großenzersdorfer Arm (Lobau).
*Scapania aspera* Bernet and M. Bernet	M	h	LC			Neuwaldegg [17].
*Scapania curta* (Mart.) Dumort.	M	n/h	LC		r	Soils at Ottakringer Wald.
*Scapania nemorea* (L.) Grolle	M	n	LC		s	Soils in the entire Sandsteinwienerwald.
*Schistidium apocarpum* (Hedw.) Bruch and Schimp.	B	n/h	LC		s	Rocks and walls in the entire urban area close to water bodies.
*Schistidium brunnescens* Limpr. subsp. *brunnescens*	B	n	VU		s	Sunny rocks in the entire urban area,
*Schistidium confertum* (Funck) Bruch and Schimp.	B	n/h	VU-R			Flyschwienerwald [14].
*Schistidium crassipilum* H.H. Blom	B	n	LC		s	Rocks in the entire urban area.
*Schistidium robustum* (Nees and Hornsch.) H.H. Blom	B	n	LC		w	Rocks in the entire urban area.
*Schistidium trichodon* (Brid.) Poelt var. *trichodon*	B	n	LC		r	Rocks at Lainzer Tiergarten.
*Sciuro-hypnum flotowianum* (Sendtn.) Ignatov and Huttunen	B	n/h	VU-R		s	Rocks and roots at Heuberg, Halterbachtal, and Rodaun.
*Sciuro-hypnum plumosum* (Hedw.) Ignatov and Huttunen	B	n/h	LC		r	Water bodies at Halterbach and Moosgraben.
*Sciuro-hypnum populeum* (Hedw.) Ignatov and Huttunen	B	n/h	LC		w	Entire urban area.
*Scorpidium cossonii* (Schimp.) Hedenäs	B	n/h	VU		r	Fen at Aumüllerwiese and Eichwiese (both Lainzer Tiergarten).
*Seligeria pusilla* (Hedw.) Bruch and Schimp.	B	n/h	LC	!	s	Rocks along small streams at Ottakringer Wald, Satzberg, Pfaffenberg, and Latisberg.
*Serpoleskea confervoides* (Brid.) Schimp.	B	n/h	LC		s	Rocks and roots in the entire Wienerwald and the Lobau.
*Solenostoma gracillimum* (Sm.) R.M. Schust.	M	n	LC		vr	Soils at Glasgraben.
*Solenostoma hyalinum* (Lyell) Mitt.	M	n/h	LC		vr	Soils at Glasgraben.
*Streblotrichum convolutum* var. *convoluta* Hedw.	B	n/h	LC		w	Soils in the entire urban area.
*Streblotrichum convolutum* var. *sardoum* (Bruch and Schimp.) Podp.	B	n	DD		r	Soils at the Technical University and Donauinsel.
*Syntrichia calcicola* J.J. Amann	B	n	NT		s	Soil at the Lobau.
*Syntrichia latifolia* (Bruch ex Hartm.) Huebener	B	n/h	LC		s	Walls at water bodies at the Schwarzenbergpark, Schottenhof, and Steinhofgründe.
*Syntrichia montana* Nees	B	n	LC		s	Kalksburg Himmelwiese.
*Syntrichia papillosa* (Wilson) Jur.	B	n/h	LC		w	Epiphyte in the entire urban area.
*Syntrichia ruraliformis* (Besch.) Cardot	B	n	VU		s	Dry soils at Himmelwiese (Kalksburg) and dry grassland at Fuchshäufl (Lobau).
*Syntrichia ruralis* (Hedw.) F. Weber and D. Mohr	B	n/h	LC		w	Soils in the entire urban area.
*Syntrichia virescens* (De Not.) Ochyra	B	n/h	LC		w	Epiphyte in the entire urban area,
*Syzygiella autumnalis* (DC.) K. Feldberg, Váňa, Hentschel and Heinrichs	M	h	NT			Pötzleinsdorf [17].
*Taxiphyllum wissgrillii* (Garov.) Wijk and Margad.	B	n/h	LC		s	Soils in the Sandsteinwienerwald.
*Tetraphis pellucida* Hedw.	B	n/h	LC		r	Deadwood at Pfaffenberg and Latisberg.
*Thamnobryum alopecurum* (L. ex Hedw.) Gangulee	B	n	LC		vr	Water bodies at Satzberg.
*Thuidium assimile* (Mitt.) A. Jaeger	B	n/h	LC		s	Soils at Steinhofgründe, Central Cemetery, and Lobau.
*Thuidium delicatulum* (Hedw.) Schimp.	B	n	LC		s	Soils in the Lobau and at Latisberg.
*Thuidium recognitum* (Hedw.) Lindb.	B	n/h	LC		s	Soils in the entire urban area.
*Thuidium tamariscinum* (Hedw.) Schimp.	B	n/h	LC		w	Soils in the entire urban area.
*Tomentypnum nitens* (Schreb. ex Hedw.) Loeske	B	n/h	VU		vr	Fen at Fasslwiese (Lainzer Tiergarten).
*Tortella densa* (Lorentz and Molendo) Crandw. and Nyholm	B	n	LC		vr	Dry grassland Kontrollerwiese (Lobau).
*Tortella fasciculata* (Culm.) Culm.	B	n	LC		r	Rocks from Rodaun to Eichkogel, Pfaffenberg, and Glasgraben.
*Tortella inclinata* (R. Hedw.) Limpr.	B	n/h	LC		w	Soils in the entire urban area.
*Tortella squarrosa* (Brid.) Limpr.	B	n/h	VU		r	Dry grasslands from Rodaun to Eichkogel and in the Lobau.
*Tortella tortuosa* (Ehrh. ex Hedw.) Limpr.	B	n/h	LC		s	Rocks in the Karbonat and Flyschwienerwald.
*Tortula acaulon* (With.) R.H. Zander var. *cuspidatum*	B	n/h	LC		w	Soils in the entire urban area.
*Tortula acaulon* (With.) R.H. Zander var. *piliferum*	B	n/h	NT		w	Dry soils and rooftops in the entire urban area.
*Tortula caucasica* Broth.	B	n/h	VU		r	Dry grasslands at Mühlleiten (Lobau) and Leopoldsberg.
*Tortula lindbergii* Broth.	B	n/h	VU		s	Dry grasslands and rooftops, Lobau, Rodaun, Central Cemetery.
*Tortula muralis* Hedw. var. *muralis*	B	n/h	LC		w	In the entire urban area.
*Tortula protobryoides* R.H. Zander	B	n/h	VU		s	Soils in the Lobau, Donauinsel, Central Cemetery and Kalksburg.
*Tortula schimperi* M.J. Cano, O. Werner and J. Guerra	B	n	DD		s	Soils in the entire Flyschwienerwald.
*Tortula subulata* Hedw.	B	n/h	LC		s	Soils in the entire urban area.
*Trichocolea tomentella* (Ehrh.) Dumort.	M	h	NT			Pötzleinsdorf [17].
*Trichodon cylindricus* (Hedw.) Schimp.	B	n/h	LC		s	Soils at the Donauinsel and in the Flyschwienerwald.
*Trichostomum crispulum* Bruch	B	n/h	LC		r	Various dry grasslands in the Lobau.
*Tritomaria exsecta* (Schmidel ex Schrad.) Schiffn. ex Loeske	B	n/h	NT		r	Wet rocks at Satzberg and Ottakringer Wald.
*Ulota crispa* (Hedw.) Brid.	B	n/h	LC		s	Epiphyte in the Flyschwienerwald close to water bodies.
*Ulota crispula* Bruch	B	n	VU			Epiphyte and deadwoods at Steinerne Lahn, and Lainzer Tiergarten.
*Warnstorfia fluitans* (L. ex Hedw.) Loeske	B	h	VU			Schönbrunn [19].
*Weissia brachycarpa* (Nees and Hornsch.) Jur.	B	n/h	NT		w	Soils in the entire urban area.
*Weissia condensa* (Voit) Lindb.	B	n	VU		vr	Dry soil at Himmelswiese (Kalksburg).
*Weissia controversa* Hedw.	B	n/h	LC		w	Soils in the in the entire urban area.
*Weissia longifolia* Mitt.	B	n/h	VU		s	Dry grasslands in the entire urban area.
*Weissia rutilans* (Hedw.) Lindb.	B	n/h	CR		vr	Soil at Heuberg.
*Weissia squarrosa* (Nees and Hornsch.) Müll.Hal.	B	n/h	CR		vr	Soil at Häuserl am Roan.
*Zygodon rupestris* Schimp. ex Lorentz	B	n	VU		vr	Epiphyte at Johannser Kogel (Lainzer Tiergarten).
